# Hepatitis Awareness Month and Testing Day — May 2017

**DOI:** 10.15585/mmwr.mm6618a1

**Published:** 2017-05-12

May 19th is National Hepatitis Testing Day in the United States to emphasize the importance of testing persons at risk for hepatitis B virus (HBV) and hepatitis C virus (HCV) infections, most of whom are unaware of their infection status. Recognizing the effectiveness of testing and other preventive and treatment measures, the National Academies of Science, Engineering, and Medicine recently set goals for the elimination of HBV and HCV as public health threats in the United States.*

HCV is the most common form of viral hepatitis in the United States and in 2013, accounted for approximately 19,000 deaths per year, a number that was greater than that of 60 other nationally notifiable infectious diseases combined (*1*). During 2010–2015, HCV incidence increased by 294% with the highest rates among young persons who inject drugs (PWID).^†^

This issue of *MMWR* includes two reports describing trends in HCV incidence and the availability of HCV prevention and treatment services that stop transmission. In the first report, only three states had comprehensive laws providing full access to HCV preventive and treatment services for PWID. The second report estimates rates of HCV infection among pregnant women in the United States and Tennessee; in the United States, HCV rates nearly doubled during 2009–2014, and in Tennessee, the rate in 2014 was approximately three times the national rate. Data from both reports emphasize the importance of viral hepatitis surveillance to identify communities at risk for HCV and public health policies that make available interventions that prevent HCV transmission and disease.

## Footnotes

* http://www.nationalacademies.org/hmd/reports/2017/national-strategy-for-the-elimination-of-hepatitis-b-and-c.aspx.

† https://www.cdc.gov/hepatitis/statistics/index.htm.
